# Early Heart Rate Recovery after a 6-min Walking Test Predicts Clinical Benefits in Patients after Percutaneous Aortic Valve Implantation

**DOI:** 10.3390/ijerph20054270

**Published:** 2023-02-28

**Authors:** Krzysztof Artur Aleksandrowicz, Katarzyna Maria Aleksandrowicz, Tomasz Grzegorz Witkowski, Michał Kosowski, Piotr Kübler, Karolina Grześkowiak, Grzegorz Piotr Golański, Damian Janusz Kulig, Maciej Filip Rachwalik, Roman Przybylski, Krzysztof Reczuch, Marcin Protasiewicz

**Affiliations:** 1 Department of Physiotherapy, Faculty of Health Sciences, Wroclaw Medical University, 50-367 Wroclaw, Poland; 2 Institute of Heart Diseases, University Hospital, 50-556 Wroclaw, Poland; 3 Department of Cardiology, Faculty of Medicine, Institute of Heart Diseases, Wroclaw Medical University, 50-367 Wroclaw, Poland; 4 Department of Cardiac Surgery and Heart Transplantation, Faculty of Medicine, Institute of Heart Diseases, Wroclaw Medical University, 50-367 Wroclaw, Poland

**Keywords:** 6 min walk test, aortic stenosis, heart rate recovery, TAVI, prognosis

## Abstract

Background: It was hypothesized that the time-appropriate return to a resting heart rate (HR) after cessation of exercise could be a marker for predicting outcomes in patients with heart failure (HF). We aimed to evaluate the prognostic value of HR recovery in functional improvement among adults with severe aortic stenosis undergoing percutaneous aortic valve implantation (TAVI). Methods: We performed a 6 min walk test (6MWT) in 93 individuals before TAVI and 3 months after the procedure. The change in walking distance was calculated. During the pre-TAVI 6MWT, we analyzed the differences between baseline HR, HR at the end of the test, and HR at the 1st, 2nd, and 3rd minute of recovery. Results: After 3 months, 6MWT distances improved by 39 ± 63 m and reached a total of 322 ± 117 m. Multiple linear regression proved the differences between HR after 2 min of recovery and baseline HR in pre-TAVI after a 6MWT was the only significant predictor of waking distance improvement during follow-up. Conclusions: Our study suggests that analysis of HR recovery after a 6MWT may be a helpful and easy parameter to assess improvements in exercise capacity after TAVI. This simple method can help to identify patients in whom no significant benefit in functional improvement can be expected despite successful valve implantation.

## 1. Introduction

Aortic stenosis (AS) caused by calcification and fibrosis of the valve leaflets leads to left ventricular outflow tract obstruction, heart failure progression and finally poor outcome. Its prevalence in developed countries is between 2–7% of patients over 65 years and its incidence increases with age. Once symptoms occur in severe AS, the prognosis is very poor without invasive treatment, with a 5-year mortality rate of 50%. According to the European Society of Cardiology and American Heart Association guidelines, transcatheter aortic valve implantation (TAVI) has become an established, less invasive treatment method than traditional surgery for severe symptomatic aortic stenosis not only in older, inoperable, or high-risk adults but also in lower-risk populations. The main randomized controlled trials exploring TAVI included patients with typical tricuspid valve anatomy; however, many real-world registries have shown also the feasibility of TAVI in bicuspid aortic valve anatomy. Parallel to native TAVI development, first transcatheter valve-in-valve TAVI procedures were introduced in 2007 and showed to be a feasible alternative to re-do surgery in symptomatic patients.

Despite the unquestionable clinical benefit, intermediate and long-term mortality rates and a lack of functional status improvement among patients undergoing TAVI are still high [1,2]. Thus, identifying those who may benefit the most from TAVI remains a priority, and different methods to select those patients are in use [3].

The 6 min walk test (6MWT) is a simple, reproducible, and easily carried out method that requires no specialized equipment or advanced training and can provide objective assessments of the functional status in a clinically impaired population. The 6 min walk test distance (6MWTD) is a widely accepted measurement of functional capacity and symptom assessment [4]. It correlates with peak oxygen consumption [5,6] and has been used as a prognostic marker in patients with heart failure (HF) [6,7,8,9,10] after aortic valve replacement [11,12,13] or after coronary revascularization [14]. Moreover, heart rate (HR) variables derived from the 6MWT, especially the differences in HR between the cessation of the exercise and after 1st or 2nd minutes of recovery have been found to be significant predictors of morbidity and mortality in patients with HF [15,16,17,18] and pulmonary hypertension [19].

It was hypothesized that the time for peak exercise HR to return to the resting HR after cessation of exercise could be a new marker for predicting outcomes in patients with HF. Accordingly, we sought to evaluate the prognostic value of HR changes during a baseline 6MWT in adults undergoing TAVI. Our primary objective was to evaluate the association between HR changes and functional improvements after the procedure.

## 2. Materials and Methods

From February 2021 to December 2021, the Institute of Heart Diseases in Wroclaw, Poland, performed a TAVI procedure in 93 patients. Patients with severe aortic stenosis were enrolled for treatment by the multidisciplinary team (Heart Team). Before TAVI, a 6MWT was performed according to the guidelines of the American Thoracic Society which describes the methodology, indications and contraindications of the test as well as practical recommendations to ensure its quality and reproducibility [20].

Patients were instructed to walk quickly for a period of 6 min or until dyspnea or muscular fatigue appeared. Prior to the start of the study, the patient was resting in a sitting position for 10 min. The walking course was 30 m in length with marks every 2 m and posts at the starting and turning places. The test was performed in a four meter wide corridor with a hard, level floor and total walking distance was recorded after 6 min HR and blood pressure was recorded with blood pressure monitor (Comfort M6 (Omron, Japan)) before, at the end of the test, and during the first, second, and third minutes of recovery. During the test the participants had to walk at a rate suitable to their condition and they were allowed to stop or slow down if necessary and asked to resume walking as soon as possible. The difference between baseline HR and HR at the end of the test was calculated (ΔHRpeak), and between baseline HR and the HR at the 1st, 2nd, and 3rd minutes of recovery (ΔHRrec_1, ΔHRrec_2, ΔHRrec_3, accordingly).

The 6MWT was repeated at the 3 month follow-up and the improvement in walking distance was calculated as the difference from baseline measurements (Δ6MWTD).

## 3. Statistical Analysis

The distribution of continuous data was tested using the Kolmogorov–Smirnov one-sample test and the Shapiro–Wilk test. The continuous variables were presented as mean ± standard deviation, whereas the categorical variables were presented as numbers and percentages. Comparisons of continuous measurements were performed using Student’s *t*-tests and chi^2^ analysis was used to compare categorical variables. The relationship between Δ6MWT and baseline parameters was tested with Pearson correlation and linear regression analysis (backward method). The study group was divided according to the presence of improvement in walking distance on the follow-up 6MWT. To test the value of baseline parameters in predicting 6MWTD improvements at 3 months post-TAVI a multivariate logistic regression analysis was performed. For all statistical tests, a *p*-value of 0.05 was considered significant. All statistical analyses were performed using SPSS version 17.0 (SPSS, Inc., Chicago, IL, USA).

## 4. Results

**Figure 1:**
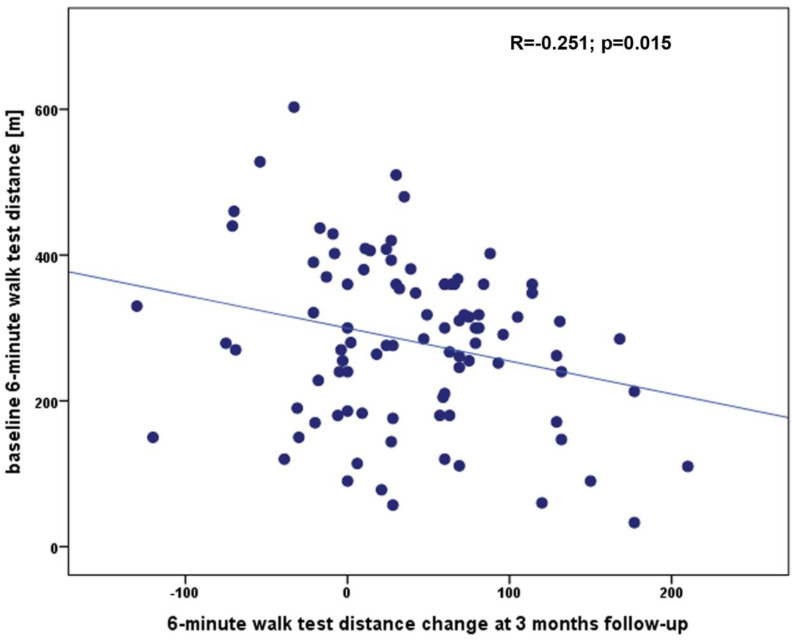
The correlation between the Δ6MWTD at the 3-month follow-up and 6 min walking test distance at baseline.

**Figure 2:**
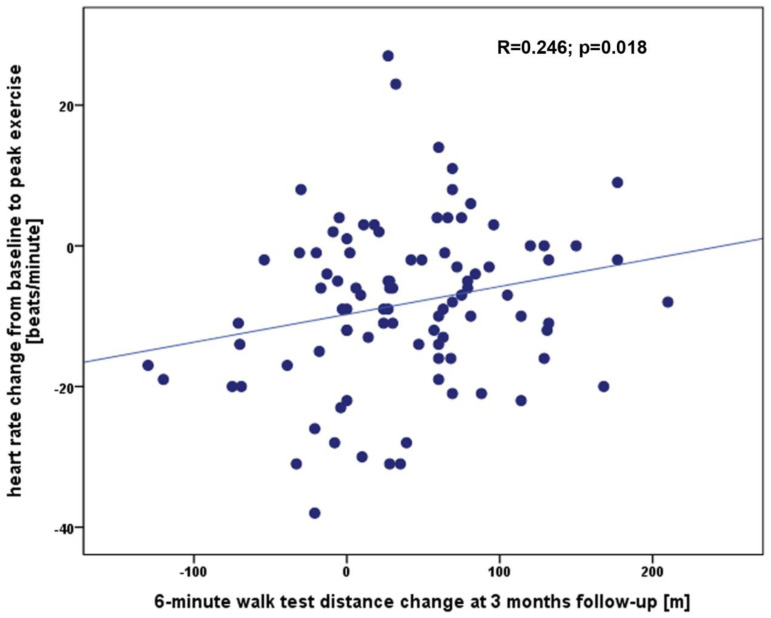
The correlation between the Δ6MWTD at the 3-month follow-up and the difference in heart rate between baseline and peak exercise.

**Figure 3:**
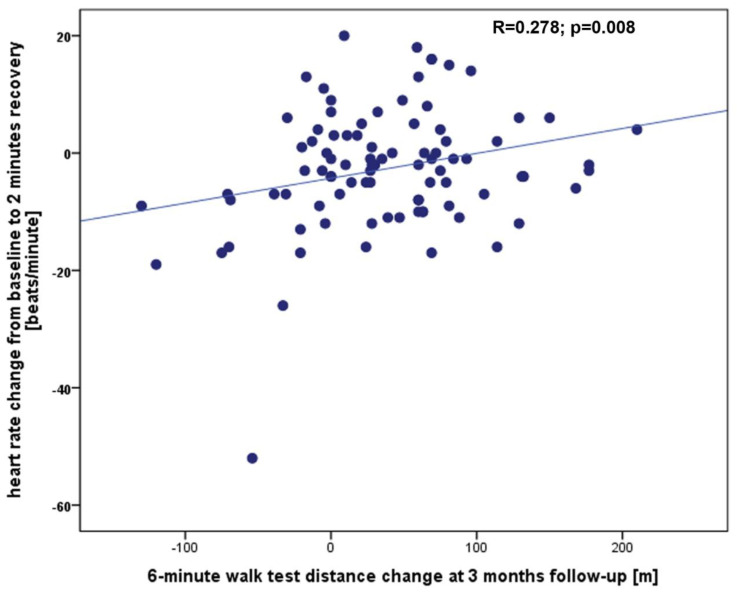
The correlation between the Δ6MWTD at the 3-month follow-up and the difference in heart rate between baseline and after 2 min of recovery.

The baseline characteristics of the study group are presented in Table 1. The 6MWT was performed in all patients and the mean 6MWTD was 281 ± 114 m, the baseline HR was 75 ± 14 beats/minute, HR at peak exercise was 83 ± 16 beats/minute, HR at 1st, 2nd and 3rd minute of recovery were 78 ± 14 beats/minute, 78 ± 16 beats/minute and 76 ± 13 beats/minute, accordingly. In all population we did not observe any significant complications during TAVI procedure which could influence walk test results in follow-up. This excellent safety profile of all valve implatations with no cases of coronary artery occlusion, annulus rupture, aortic dissection, stroke, major bleeding, major vascular complications or procedural death is an important finding of our research. At 3 months, the 6MWT was repeated and the 6MWTD improved to 322 ± 117 m resulting in a Δ6MWTD of 39 ± 63 m. There was no correlation between Δ6MWTD and clinical parameters such as age, body mass index, left ventricle ejection fraction, hemoglobin, and creatinine (for all *p* > 0.05), but a statistically significant correlation was found between Δ6MWTD and baseline 6MWTD (Figure 1), ΔHRpeak (Figure 2), and ΔHRrec_2 (Figure 3). The remaining 6MWT parameters showed no correlation to Δ6MWTD.

A multiple linear regression backward method was used to test if the baseline 6MWTD, ΔHRpeak, and ΔHRrec_2 significantly predicted Δ6MWTD, of which ΔHRrec_2 was the only significant predictor (Table 2).

In 28 patients (30%) there was no improvement in 6MWTD at the 3-month follow-up after TAVI. In fact, we observed a decrease of 6MWTD above 30 m in this subgroup. The comparison of clinical and 6MWT parameters in patients with and without improvement in 6MWTD is presented in Table 3.

Only the creatinine level before TAVI, HR at peak exercise and HR at 1st, 2nd and 3rd minute of recovery differed significantly between patients with and without improvements in 6MWTD. All parameters were evaluated using a multivariate logistic regression analysis (backward method) to check their relationship with the 6MWTD improvement at 3 months after TAVI. It was found that only ΔHRrec_2 remained in the model with an odds ratio of 1.068 (Confidence Interval 1.012–1.128), *p* = 0.017, χ^2^ of the model = 7.21, *p* = 0.007.

**Figure 4:**
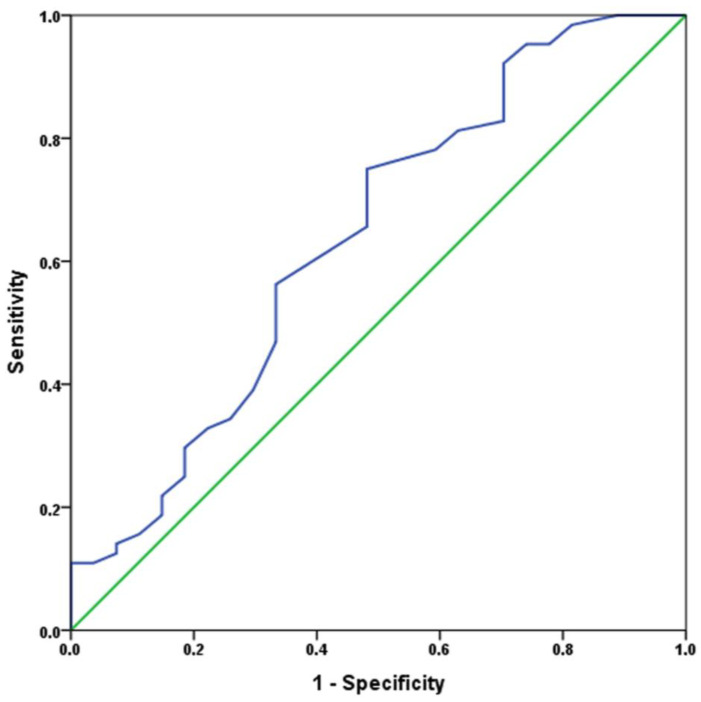
The area under the curve is 0.64 (CI 0.51–0.77), *p* = 0.037.

Receiver operating characteristic analysis of the difference in HR at 2nd minute of recovery and baseline (ΔHRrec_2) for 6MWTD after transcatheter aortic valve replacement (Figure 4). The optimal cut-off value ΔHRrec_2 to predict 6MWTD improvement was determined by the highest sum of sensitivity (56%) and specificity (73%) which in this case was −2 beats/minute.

## 5. Discussion

Previous studies have shown that not all patients after a successful TAVI procedure will improve in the 6MWT during follow-up [21]. Our study was designed to evaluate the hypothesis that HR recovery after 6MWT is a prognostic factor for improvement in the test results.

The impaired chronotropic response to exercise was shown to be a predictor of mortality among patients with known or suspected coronary disease. Lauer et al. showed that in patients referred for stress testing with thallium imaging, chronotropic incompetence was predictive of all-cause mortality in 2 years of follow-up. Authors concluded that incorporation of chronotropic response into the interpretation of stress thallium studies may improve significantly its prognostic power [22]. Andrare et al. aimed to determine whether the time for peak exercise heart rate to return to resting heart rate after the 6MWT may be important to predict cardiac events in patients with heart failure (HF). Their study showed that a HR recovery time above 3 min after the 6MWT is an independent risk factor for cardiac events in HF patients [23]. The study of Swigris et al. proved that abnormal HR recovery is associated with right heart catheterization-confirmed pulmonary hypertension [19].

The results of the current study show that HR recovery after the 6MWT may be a predictor of improved exercise capacity in TAVI patients at 3 months. We observed that patients with appropriate HR recovery benefited from the procedure in terms of improved walking distance during the follow-up period. The optimal cut-off value to predict post-TAVI 6MWTD improvement was −2 beats/minute after 2 min of recovery concerning the baseline HR in the pre-TAVI test.

Altisent et al. showed that the absence of an improvement in physical performance at 6 months post-TAVI is an independent predictor of mortality and adverse cardiovascular outcomes [24]. Mok et. al. demonstrated an association between 6MWTD and all-cause mortality among 212 TAVI recipients who were able to perform the test (HR 1.08, 95% CI 1.04 to 1.13 for each 10 m decrease in 6MWTD) [13].

Thus, our results may have important clinical significance and suggest that systematic implementation of exercise capacity and HR recovery assessments in pre-TAVI patients may help to improve patient risk stratification. Close to 30% of patients who underwent TAVI in our study did not improve their exercise capacity after 3 months despite a successful procedure. This result is very similar to previous observations [22,24]. Also, Bagur, who performed 6 min walk tests in 64 TAVI patients at baseline and 6 months after procedure demonstrated that although overall 6MWTD increased, 25% of subjects did not improve their 6MWTD at follow-up compared with initial result [25]. Identification of this group before valve implantation may be helpful to specify who will require especially intensive medical follow-up assessments or even more individualized cardiac rehabilitation.

The mean 6MWTD at baseline and follow-up in our cohort was relatively higher compared to other studies like the PARTNER trial where distances achieved by study population were considerably below the normal limits [26]. This can be explained by the fact that the majority of our patients (82%) were intermediate-risk patients, they were also younger, while the PARTNER trial included a more burdened population. However, similarly to this population and our study, patients with short walking distances at baseline experienced a higher improvement at their 3 month 6MWT. Whereas the fast walkers at baseline did not improve or experienced a modest decrease in 6MWTD. It is interesting to note that the relationship between older age and female sex with impaired improvements in 6MWTD has been observed previously [27]. However, our analysis does not reaffirm the significance of these adverse prognostic factors, which may be surprising.

## 6. Conclusions

In conclusion, our study suggests that HR recovery after 6MWT may be a helpful and clinically easy-to-assess parameter to predict exercise capacity improvement after TAVI. This simple method may help to identify patients in whom no significant benefit can be expected and those with a worse prognosis requiring more personalized medical care.

There are important limitations to our study. First, though this is a prospective observational study, a small number of patients were included in the analysis. The lack of randomization and the absence of data-gathering monitoring—including self-reported 6MWT outcomes—constitute significant limitations of our research. Consequently, our findings should be considered hypothesis-generating only. Second, the proposed analysis can be performed only in patients able to walk and undergo a 6MWT. Finally, different markers of frailty were not analyzed in our study, as such the incremental prognostic value of HR recovery after 6MWT over that of frailty markers cannot be assessed.

## Figures and Tables

**Table 1:** Baseline characteristics of patients with severe aortic stenosis before the transcatheter aortic valve implantation procedure.

Age (years)	78 ± 6
Men [*n*, (%)]	44 (47)
Body mass index (kg/m^2^)	29.5 ± 5.6
Prior myocardial infarction [*n*, (%)]	39 (42)
Diabetes mellitus [*n*, (%)]	36 (39)
Chronic heart failure [*n*, (%)]	35 (38)
Chronic obstructive pulmonary disease [*n*, (%)]	6 (7)
Chronic kidney disease [*n*, (%)]	39 (42)
Creatinine (mg%)	1.5 ± 1.6
Hemoglobin (g%)	12.6 ± 1.6
Left ventricular ejection fraction (%)	57 ± 10

**Table 2:** Multiple linear regression (backwards method) model for predictors of changes in 6 min walking test distance from baseline to 3 the month follow-up after transcatheter aortic valve replacement.

	B	95% Confidence Interval	*p*
CONSTANT	70.5	35.8–105.1	
Baseline 6-min walk test distance	0.18	−0.22–0.01	0.088
Heart rate change between baseline and 2 min recovery	0.22	0.08–2.60	0.038

**Table 3:** Comparison of clinical and 6 min walking test parameters in patients with and without walking distance improvement after transcatheter aortic valve replacement procedure.

	Improvement	No Improvement	*p*
Age (years)	78 ± 7	79 ± 6	0.357
Men [*n* (%)]	33 (50)	11 (39)	0.374
Body mass index (kg/m^2^)	29.2 ± 6.6	30.3 ± 4.9	0.316
Prior myocardial infarction [*n*, (%)]	24 (36)	15 (54)	0.121
Diabetes mellitus [*n*, (%)]	24 (36)	13 (46)	0.361
Chronic heart failure [*n*, (%)]	27 (41)	8 (29)	0.258
Chronic obstructive pulmonary disease [*n*, (%)]	5 (8)	1 (4)	0.468
Chronic kidney disease [*n*, (%)]	26 (39)	13 (46)	0.527
Creatinine (mg%)	1.3 ± 1.1	1.8 ± 2.3	0.028
Hemoglobin (g%)	12.7 ± 1.6	12.3 ± 1.8	0.636
Left ventricular ejection fraction (%)	56 ± 11	59 ± 8	0.060
NYHA class before TAVI	2.7 ± 0.3	2.6 ± 0.2	0.867
NYHA class after TAVI	2.1 ± 0.3	2.5 ± 0.2	0.277
6MWTD baseline (m)	275.1 ± 106.9	299.6 ± 128.7	0.172
6MWTD follow-up (m)	343 ± 101.6	268.6 ± 129.8	0.078
Δ6MWTD (m)	68.6 ± 46.8	−31.0 ± 35.7	0.187
HR before 6MWT (beats/minute)	74 ± 13	78 ± 15	0.176
HR at peak exercise (beats/minute)	80 ± 13	90 ± 20	0.016
HR at 1st minute of recovery (beats/minute)	75 ± 13	84 ± 16	0.016
HR at 2nd minute of recovery (beats/minute)	75 ± 13	84 ± 19	0.006
HR at 3rd minute of recovery (beats/minute)	74 ± 13	81 ± 13	0.012
Δ HRpeak (beats/minute)	−6 ± 11	−12 ± 11	0.610
ΔHRrec_1 (beats/minute)	−1 ± 10	−6 ± 9	0.760
ΔHRrec_2 (beats/minute)	−1 ± 8	−6 ± 13	0.073
ΔHRrec_3 (beats/minute)	0 ± 9	−3 ± 9	0.950

6MWTD—6 minute walk test distance; Δ6MWTD—the difference between 6MWTD baseline and 6MWTD follow-up; ΔHRpeak—heart rate difference between baseline and peak exercise; ΔHRrec_1—heart rate difference between baseline and 1st minute of recovery; ΔHRrec_2—heart rate difference between baseline and 2nd minute of recovery; ΔHRrec_3—heart rate difference between baseline and 3rd minute of recovery; NYHA—New York Heart Association.

## Data Availability

The datasets generated and/or analysed during the current study are available from the corresponding author upon reasonable request.
